# Supplementary material for: Structure Based Affinity Maturation and Characterizing of SARS-CoV Antibody CR3022 against SARS-CoV-2 by Computational and Experimental Approaches
Source: Viruses. 2022 Jan 19;14(2):186. doi: 10.3390/v14020186 (PMC8875849; doi:10.3390/v14020186)

viruses

MDPI

Figure S1. The binding kinetics and rate constants of SARS-CoV-RBD to single-site mutated antibodies measured by surface plasmon resonance (SPR).

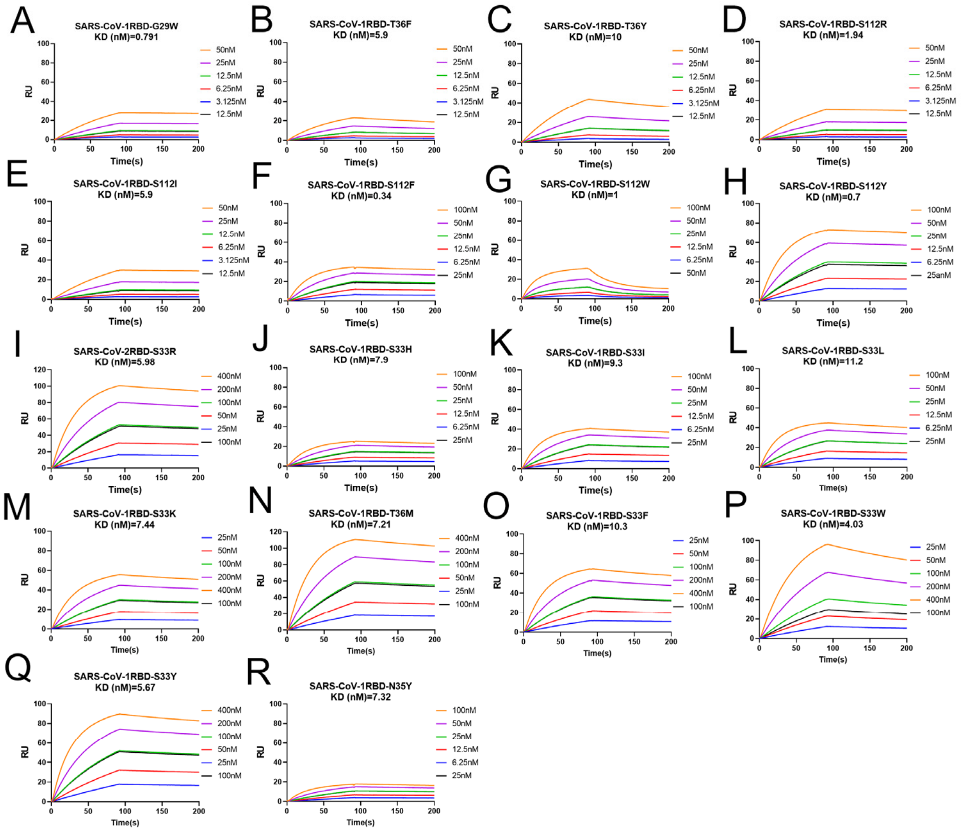

Figure S2. The binding kinetics and rate constants of SARS-CoV-2-RBD to single-site mutated antibodies measured by surface plasmon resonance (SPR).

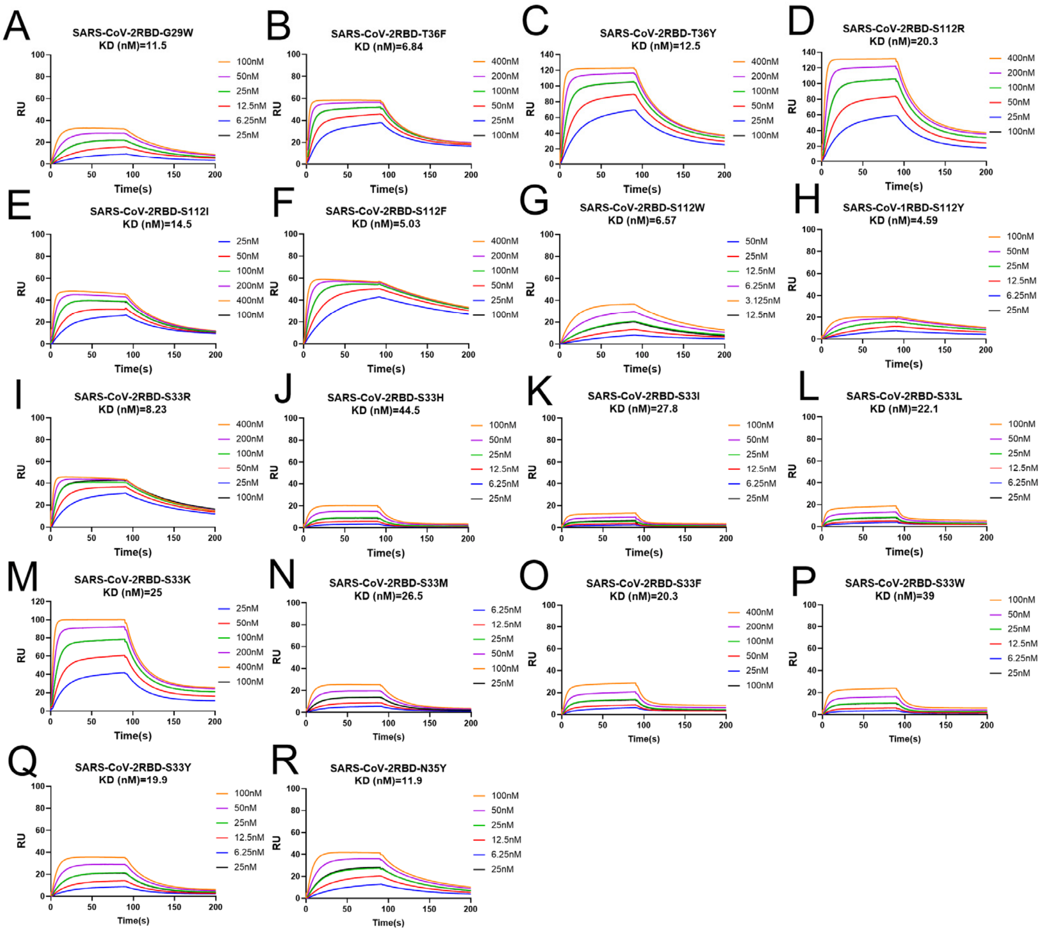

Supplement: Supplementary file 1 [file viruses-14-00186-s001.zip › viruses-1531796-supplementary.pdf]
